# Draft Genome Sequence of Exopolysaccharide-Producing Cyanobacterium Aphanocapsa montana BDHKU 210001

**DOI:** 10.1128/genomeA.00057-15

**Published:** 2015-03-05

**Authors:** Sourav Bhattacharyya, Mathu Malar Chandrababunaidu, Deeya Sen, Arijit Panda, Arpita Ghorai, Sushma Bhan, Neha Sanghi, Sucheta Tripathy

**Affiliations:** Structural Biology and Bioinformatics Division, CSIR, Indian Institute of Chemical Biology, Kolkata, West Bengal, India

## Abstract

We report for the first time the draft genome sequence of Aphanocapsa montana BDHKU 210001, a halotolerant cyanobacterium isolated from India. This is a marine exopolysaccharide (EPS)-producing cyanobacterium. The genome of this species is assembled into 11.50 million bases, with 296 scaffolds carrying approximately 7,296 protein-coding genes.

## GENOME ANNOUNCEMENT

Marine cyanobacteria are a major production source of various bioactive, antifungal, antibacterial, and anticancer elements having immense economic value (1). Several of these species also have protease activities (2) and oil-degrading and bioremediation properties (3). It has been established that marine cyanobacteria produce different types of metabolites through nonribosomal pathways that are known to have medicinal properties (4). Several cyanobacteria belonging to the *Chroococcales* produce exopolysaccharides (EPS) that can be used as a heavy-metal chelating agent (5).

Aphanocapsa spp. produce an antifungal element, cyanopeptolin CB071, which acts as a trypsin inhibitor (6). Several Aphanocapsa spp. are cyanobionts with marine sponges and fix nitrogen for their host (7). Aphanocapsa montana BDHKU 210001, which belongs to the order *Chroococcales*, was initially isolated and maintained at the National Facility For Marine Cyanobacteria, Tiruchirappalli, India. The cultures were grown in ASN medium (8) and maintained in a 16-/8-h light-dark photo period, without shaking and at room temperature (~28°C), at our facility at the Indian Institute of Chemical Biology, Kolkata, India. The genomic DNA was extracted and purified using the UniFlex bacterial isolation kit (Genei, USA), yielding a final concentration of 29.6 ng/µl genomic DNA. A paired-end library with a 300-base insert size and a mate-pair library with a 3-kb insert size were prepared for sequencing. Whole-genome sequencing was carried out using an Illumina HiSeq platform generating approximately 19.74 million reads, with an average read length of 151 bases from the paired-end library. For the mate-pair library, 5.4 million reads, with an average read length of 101 bases, were generated. The sequences were cleaned prior to assembly using SGA and TagDust from the A5 pipeline (9). The sequences were finally assembled from 250× coverage paired-end and 39× coverage mate-pair data using the Allpaths-LG-49856 (10) assembler. The final draft genome, having 296 scaffolds and 11.50 Mb, with an *N*_50_ value of 146,514, was assembled from this data. The total G+C content of the genome was calculated to be 55.4%, and the largest and smallest scaffolds were 5,058,360 bp and 4,530 bp, respectively. The genome was annotated using the PGAAP from the NCBI (http://www.ncbi.nlm.nih.gov/genomes/static/Pipeline.html).

The annotation revealed a total of 8,867 genes, out of which 1,315 are pseudogenes and 7,296 are protein-coding genes. A total of 54 tRNA genes, 1 noncoding RNA gene, and 10 clustered regularly interspaced short palindromic repeats (CRISPRs) in CRISPR-Cas were predicted (11).

Several interesting genes were found in this organism, including the nif cassette for nitrogen fixation, many copies of the quorum-sensing gene *cheY*, few alcohol dehydrogenases, etc. Genes conferring resistance to colicin ExbB-ExbD are also found in this organism, with only 45% identity with the closest cyanobacterium, the Oscillatoria ExbB-ExbD complex.

### Nucleotide sequence accession number.

The whole-genome and annotation data for A. montana BDHKM 210001 have been submitted to GenBank under the accession no. JTJD00000000.
